# Impact of Early Feeding: Metagenomics Analysis of the Infant Gut Microbiome

**DOI:** 10.3389/fcimb.2022.816601

**Published:** 2022-03-04

**Authors:** Matthew D. Di Guglielmo, Karl R. Franke, Alan Robbins, Erin L. Crowgey

**Affiliations:** ^1^ Division of General Academic Pediatrics, Department of Pediatrics, Nemours Children’s Health, Wilmington, DE, United States and Sidney Kimmel Medical College at Thomas Jefferson University, Philadelphia, PA, United States; ^2^ Biomedical Research Department, Nemours Children’s Health, Wilmington, DE, United States

**Keywords:** metagenomics, next generation sequencing, gut microbiome, whole genome, breast-feeding, infants

## Abstract

**Background:**

Different feeding regimens in infancy alter the gastrointestinal (gut) microbial environment. The fecal microbiota in turn influences gastrointestinal homeostasis including metabolism, immune function, and extra-/intra-intestinal signaling. Advances in next generation sequencing (NGS) have enhanced our ability to study the gut microbiome of breast-fed (BF) and formula-fed (FF) infants with a data-driven hypothesis approach.

**Methods:**

Next generation sequencing libraries were constructed from fecal samples of BF (n=24) and FF (n=10) infants and sequenced on an Illumina HiSeq 2500. Taxonomic classification of the NGS data was performed using the Sunbeam/Kraken pipeline and a functional analysis at the gene level was performed using publicly available algorithms, including BLAST, and custom scripts. Differentially represented genera, genes, and NCBI Clusters of Orthologous Genes (COG) were determined between cohorts using count data and R (statistical packages edgeR and DESeq2).

**Results:**

Thirty-nine genera were found to be differentially represented between the BF and FF cohorts (FDR ≤ 0.01) including *Parabacteroides*, *Enterococcus*, *Haemophilus*, *Gardnerella*, and *Staphylococcus*. A Welch t-test of the Shannon diversity index for BF and FF samples approached significance (*p*=0.061). Bray-Curtis and Jaccard distance analyses demonstrated clustering and overlap in each analysis. Sixty COGs were significantly overrepresented and those most significantly represented in BF vs. FF samples showed dichotomy of categories representing gene functions. Over 1,700 genes were found to be differentially represented (abundance) between the BF and FF cohorts.

**Conclusions:**

Fecal samples analyzed from BF and FF infants demonstrated differences in microbiota genera. The BF cohort includes greater presence of beneficial genus *Bifidobacterium.* Several genes were identified as present at different abundances between cohorts indicating differences in functional pathways such as cellular defense mechanisms and carbohydrate metabolism influenced by feeding. Confirmation of gene level NGS data *via* PCR and electrophoresis analysis revealed distinct differences in gene abundances associated with important biologic pathways.

## 1 Introduction

Early dietary content is an important consideration in the long-term development of immunologic, metabolic, and many chronic disorders (Singhal and Lanigan, 2007; Cox et al., 2014; Rodriguez et al., 2015; Clapp et al., 2017; Davis et al., 2017; Davis et al., 2020; Turroni et al., 2020; Sarkar et al., 2021). Analyzing the infant fecal microbiome to understand the effects on the gastrointestinal (gut) microbiota conferred by early feeding/diet could help elucidate the mechanism underlying the development of these phenotypes. Next generation sequencing (NGS) is a technique that enables deep probing of both meta-taxonomy of the gut flora as well as the metagenomics signature of the microbiome. Dietary differences may have a long-lasting effect on the gut microbiome by impacting the composition and biological functions of the organisms present. This study seeks to contrast taxonomic variation between breast-fed (BF) and formula-fed (FF) infants at the genera level to identify orthologous gene clusters that may be in differential abundance between the cohorts. Additionally, the study attempts to characterize the metagenome composition and differences between BF and FF cohorts.

Previous studies, including work from our laboratory, demonstrate differences in the gut microbiome of BF versus FF infants (Lee et al., 2015; Schwartz et al., 2012; Bäckhed et al., 2015; Baumann-Dudenhoeffer et al., 2018; Stewart et al., 2018; Di Guglielmo et al., 2019). These studies highlight species diversity between differently fed infants using a 16S ribosomal RNA analysis (Schwartz et al., 2012; Bäckhed et al., 2015; Lee et al., 2015) and identify key genera abundance dissimilarities between FF and BF very young infants. Key findings include a significant predominance of the *Bifidobacterium* genus in BF infants and more abundant *Enterococcus* and *Escherichia* genera in FF samples. Additional metagenomic analysis using a shotgun approach, versus a 16S ribosomal RNA approach, indicates a diversity of gut microbiota at both the genera and gene level (Baumann-Dudenhoeffer et al., 2018; Stewart et al., 2018; Di Guglielmo et al., 2019).

Formula feeding influences the persistence of a more diverse, but not necessarily beneficial, gut microbiota (Davis et al., 2020). Prior work in our laboratory demonstrate differential levels of bacterial genes in each cohort showing a relative lower abundance of seven genes in the FF infants contrasted with 364 genes with a higher relative abundance. The most notable change in gene abundance is the lack of one gene (CRISPR-Cas9) in FF fecal samples. CRISPR-Cas9 is a key component of bacterial cellular defense mechanisms for protecting against both pathogenic mutations and antibiotic resistance. Overall, the specific genes in question suggest a biological explanation for gut microbiota acting differently on the intestinal epithelium in the development of pathogenic strains, drug resistance, and biofilm formation.

Other studies demonstrate that different short-chain fatty acids dominate in BF versus FF infant fecal samples (Fukuda et al., 2012; den Besten et al., 2013; Cox et al., 2014). These metabolic differences may reflect the gut microbiota composition that generates these small molecules and the downstream effects on host gastrointestinal, immunologic, and neurologic functions. Further analysis of these microbiome differences is required to expand our understanding of how diet early in life impacts the gut microbiota and microbiome.

Research on the infant microbiome has demonstrated both pliability as well as susceptibility to external influence (Singhal and Lanigan, 2007; Cox et al., 2014; Davis et al., 2020; Sarkar et al., 2021). Particular microbiota species are known to be dominant in BF infants (Pannaraj et al., 2017). Studies of the genus *Bifidobacterium* suggest a critical symbiotic role of human milk oligosaccharides in breast milk and the genus of organisms that metabolize them (Sela et al., 2008; Bode, 2012; Ruiz-Moyano et al., 2013; Garrido et al., 2015; Lewis et al., 2015). The long-term health implications of microbiome changes may not be subtle. If a more pro-inflammatory or pro-pathogenic environment is fostered in the gut of young infants due to dietary influences that change micro-organisms, gene expression, and carbohydrate metabolism, individuals may develop increased immune disorders (Schwartz et al., 2012), the need for broad-spectrum antibiotics (Taft et al., 2018; Casaburi et al., 2019), and gastrointestinal and neurologic disorders (Clapp et al., 2017; Kang et al., 2017; Kang et al., 2019). Clinical care of young patients could be directed to protect the beneficial gut microflora and focus efforts on influencing it early in life when the microbiome is still adaptable; long-term benefits on adolescent and adult health may follow (Singhal and Lanigan, 2007; Cox et al., 2014; Davis et al., 2020; Sarkar et al., 2021).

To better understand the importance of both taxonomy and differential gene abundance, the present study employs whole-genome fecal metagenomic next-generation sequencing and a computational pipeline previously used in our laboratory (Di Guglielmo et al., 2019). The goal of this manuscript is to expand the prior analysis in size and interpretation of the metagenomics data and create a refined and more accurate picture of the metataxonomic profile of the cohorts studied. Each Clusters of Orthologous Genes (COG) pattern that emerges from the analysis of gene abundance can suggest functional roles. Coupling taxonomic differences with gene abundance as a proxy for functional and biological significance allows a hypotheses for testing future interventions that could modify the gut microbiota in a beneficial way for the health of patients.

## 2 Materials and Methods

### 2.1 Subject Enrollment

The study was approved by the Nemours Institutional Review Board #1458092 and #822736. Parental permission was obtained from each infant’s parent or guardian. The FASTQ data will be deposited online at SRA (or equivalent). Thirty-four healthy term infants between 5 days and 100 days of age who were exclusively breast fed (n=24; age range 5-95 days) or formula fed (n=10; age range 10-100 days) were recruited. Infants were excluded if they had any other sources of nutrition, dietary restrictions (e.g., hypoallergenic formula), consumed higher density formula (>20 calories/ounce), had exposure to antibiotics, or had any gastrointestinal infection or disease that affected the integrity of the intestinal mucosa. Fecal samples and clinical data on infants were collected, including demographic information, maternal and paternal age (years) at infant’s birth, maternal and paternal height and weight, delivery method, maternal antibiotic use (breast-feeding mothers only), and maternal over the counter or prescription medications taken during pregnancy.

### 2.2 Sample Collection

Soiled diapers were sampled within 12 hours of defecation. Stool was collected by application of two duplicate swabs (Copan Diagnostics, Murrieta, CA) for metagenomics sequencing. The containers were placed immediately into a dry ice ethanol bath and then transferred to a -80°C freezer until processing.

### 2.3 DNA Extraction and Sequencing

DNA extraction and sequencing were completed at the Microbiome Center at the Children’s Hospital of Philadelphia. DNA was extracted from samples using the DNeasy PowerSoil kit using the manufacturer’s instructions (Qiagen, Germantown, MD). Libraries were generated from 1 ng of DNA using the NexteraXT kit (Illumina, San Diego, CA) and sequenced on the Illumina HiSeq 2500 using 2x125bp chemistry in high output mode. Extraction controls (no template) and DNA free water were included to empirically assess environmental and reagent contamination. Laboratory-generated mock communities consisting of DNA from *Vibrio campbellii*, *Cryptococcus diffluens*, and Lambda phage were included as positive controls.

### 2.4 Bioinformatics Analysis

Microbiome NGS library analysis was performed using the same pipeline as our previous study (Di Guglielmo et al., 2019) with a few modifications. Briefly, the “QC” part of the Sunbeam pipeline (Clarke et al., 2019) was used to remove adapters, human, and PhiX contamination before the Sunbeam “Classify” portion used a Kraken1 (Wood and Salzberg, 2014) database built on October 23, 2018 to classify the decontaminated reads. Trimmed mean of M-values normalization and statistical testing were performed with edgeR (Robinson et al., 2010) and DESeq2 (Love et al., 2014) to calculate statistically significant differentially represented genera.

Shannon diversity indexes were calculated *via* the VEGAN R package (Dixon, 2003). Metagenome construction was done *via* MEGAHIT (Li et al., 2015) using concatenated decontaminated FASTQ files as inputs. Prodigal (Hyatt et al., 2010) and NCBI COGs (Tatusov et al., 1997) were used for gene prediction and annotation. STAR (Dobin et al., 2013) was used to map individual samples’ decontaminated FASTQ files to the metagenome. RSEM (Li and Dewey, 2011) was used to count the number of reads mapping to unique genes, and a custom script was used to count the number of reads mapping to unique National Center for Biotechnology Information COGs. The edgeR and DESeq2 packages were used to calculate statistically significant differentially represented genes and COGs between cohorts. Heatmaps were generated using the pheatmap R package. NGS read depths are listed in **Supplemental Table 1**.

### 2.5 Direct PCR Validation

Specific genes that were more abundant or less abundant in either cohort, or that mapped to specific COGs having statistically significant differences in representation between cohorts, were subjected to validation using primers specific for each gene. The PCRs were performed using Takara (Takara Bio Inc., Shiga, Japan) 50X Titanium Taq DNA polymerase. Each 25 µl PCR reaction contained 2.5 µl 10X Takara Taq buffer (S1793), 0.5 µl 10 mM dNTP mix (Sigma cat# D7295), 1 µl 10 µM oligos IDT, 0.25 µl 50X Takara Titanium Taq DNA polymerase (S1792), 1 µl DNA (extracted from fecal samples), and 19.75 µl H_2_O. The PCR conditions are 5 minutes at 95°C for 1 cycle, 30 seconds at 95°C for 35 cycles, 30 seconds at 66°C for 35 cycles, 30 seconds at 72°C for 35 cycles, and 7 minutes at 72°C for 1 cycle. Five microliters of each PCR were run on a 3% NuSieve agarose gel in 1X TAE buffer and visualized using ethidium bromide staining. NGS reads for the target genes are displayed in **Supplemental Table 2**; qPCR primers used for the target genes are listed in **Supplemental Table 3**.

## 3 Results

### 3.1 Patient Demographics

Thirty-four subjects were enrolled, and duplicate fecal samples were processed for each subject. **Table 1** details demographic information about subjects. Twenty-four infants were exclusively breast fed and 10 were exclusively formula fed. No subjects were exposed to antibiotics. There were no statistical differences noted in the demographic data (**Table 1**) between the BF and FF cohort except for delivery method (*p*-value <0.01).

**Table 1:** Subject Demographic and Anthropometric Data.

	Breast Fed (n = 24)	Formula Fed (n = 10)	P
Sex, Female	54%	20%	0.07
Age, days (mean, SD)	48.4, 32.4	53.7, 24.3	0.59
Age, days (median, IQR)	37.5, 63	54, 18.8	
Race, Caucasian	67%	70%	0.85
Ethnicity, Non-Hispanic	79%	80%	0.96
Delivery method, SVD	79%	30%	<0.01
Birth weight, grams (mean, SD)	3317, 407	3335, 221	0.87
Enrollment weight, grams (mean, SD)	4519, 996	4554, 921	0.92
Maternal age, years (mean, SD)	30.9, 4.7	31.1, 6.2	0.92
Paternal age, years (mean, SD)	32.6, 6.1	33.4, 7.8	0.78
Maternal BMI, kg/m2 (mean, SD)	27.6, 7.5	26.6, 3.4	0.54
Maternal pre-pregnancy BMI, kg/m2 (mean, SD)	26.4, 7.9	26.3, 5.1	0.97
Paternal BMI, kg/m2 (mean, SD)	27.7, 8.2	28.7, 7.3	0.75

p values for categorical variables were calculated using Chi squared test, p values for numerical variables were calculated using Student’s t-test. SD, standard deviation; IQR, interquartile range; SVD, spontaneous vaginal delivery; BMI, body mass index.

### 3.2 Metagenomic Sequencing Beta-Diversity and Genera Analysis

**Figure 1:**
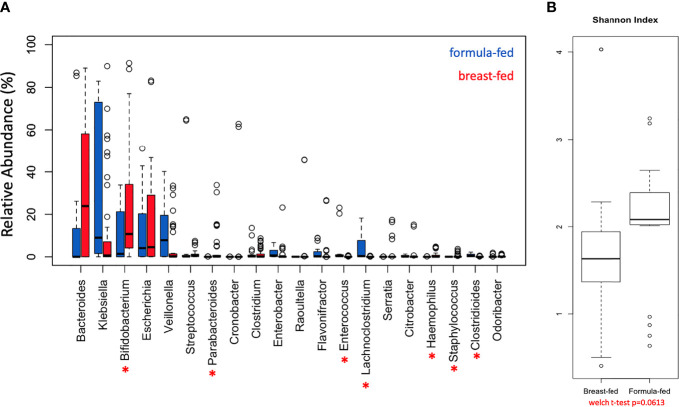
Differentially Represented Genera. Distribution of genera identified in the gut microbiome of breast-fed and formula-fed infants. **(A)** Boxplot of the topmost abundant genera in breast-fed infants (red boxes) and the topmost abundant genera in formula-fed infants (blue boxes). The red asterisks represent the genera that were statistically different between the breast-fed and formula-fed cohorts. The y-axis represents phylogenetic abundance (percentage), and each genus is represented on the x-axis. Asterisk (*) represents some of the 39 genera in total that were differentially represented with FDR ≤ 0.01. **(B)** Shannon diversity index comparing breast-fed and formula-fed infants.

The genera abundance was analyzed per cohort, FF and BF, and plotted as relative % abundance (**Supplemental Figure 1A**). There were similarities noted in presence/absence of genera, including *Bacteroides, Klebsiella, Bifidobacterium, Escherichia*, and *Veillonella*; however, differences in abundances were noted between the cohorts. Genera abundances were also plotted per sample (**Supplemental Figure 1B**) and differences within a cohort were noted. There were consistent patterns between cohorts, with seven of the 20 most abundant genera (**Figure 1A**) having abundance differences that were statistically significant (asterisks). The distribution of the Shannon diversity index was examined per each cohort and plotted as box-whisker plots (**Figure 1B**), and a wider distribution was noted in the BF cohort compared with the FF cohort, but the comparison did not reach significance (Welch’s t-test *p*-value = 0.0613). In total, 39 genera exhibited differences in abundance that were statistically significant between the FF and BF cohorts (*p*-value < 0.05 with an FDR ≤ 0.01 for both edgeR and DESeq2) (**Supplemental Table 4**). Consistent with our previous metagenomics work studying the metagenome of FF versus BF infants (Di Guglielmo et al., 2019), *Parabacteroides, Haemophilus, Enterococcus, Staphylococcus*, and *Phietavirus* were differentially represented between the cohorts (**Supplemental Table 4**, bold). To determine consistency of the bio-replicates in the BF and FF cohorts, a Bray-Curtis dissimilarity and Jaccard distance principal coordinate analysis were conducted (**Supplemental Figure 2**). The Bray-Curtis plot (**Supplemental Figure 2A**) demonstrated consistency between the bio-replicates (circle and triangles) using species abundance data, and the Jaccard distance plot (**Supplemental Figure 2B**) demonstrated consistency between bio-replicates (circle and triangles) using binary (plus/minus) species data.

### 3.3 Gene Level Analysis

To determine potential functional differences in the metagenomes between the FF and BF cohorts, a gene level analysis was conducted by creating a co-assembly of all the sequencing data that was then utilized to map and annotate individual level sample data. In total, 1,734 genes (annotated *via* Prodigal) were identified as statistically different in abundance (count data) between the FF and BF cohorts (**Supplemental Table 5**). Genes that were higher in abundance in FF samples included functional annotations (NCBI COG) such as DNA segregation ATPase, NADPH ubiquinone oxidoreductase subunit 4, DNA topoisomerase IA, and a sugar phosphate permease (**Supplemental Table 5**, bold). Genes that were higher in abundance in BF samples included functional annotations such as retron-type reverse transcriptase, type IV secretory pathway (relaxase), beta-galactosidase/beta-glucuronidase, and OmpR family (**Supplemental Table 5**, underline).

To determine if a higher-level analysis of the COGs would increase the interpretation of the functional annotations, COGs were collapsed/rolled up based on their functional hierarchy. The abundance differences of 60 COGs were identified as statistically significant when comparing the FF cohort with the BF cohort (**Table 2**). Using COG count level data, genes and COGs were clustered and visualized using a heatmap approach (**Supplemental Figure 3**).

**Table 2:** Significant Abundance Differences of COG Between Formula-fed and Breast-fed infants.

COG	Description	Category	logFC	logCPM	FDR-edgeR	Padj- DESeq2
**COG3549**	Plasmid maintenance system killer protein	Defense mechanisms	-6.303	4.665	1.47E-03	1.52E-03
**COG3914**	Predicted O-linked N-acetylglucosamine transferase, SPINDLY family	Posttranslational modification, protein turnover, chaperones	-5.142	4.459	3.90E-04	3.93E-08
**COG4115**	Toxin component of the Txe-Axe toxin-antitoxin module, Txe/YoeB family	Defense mechanisms	-4.391	9.353	6.97E-04	1.33E-06
**COG5527**	Protein involved in initiation of plasmid replication	Mobilome: prophages, transposons	-4.302	12.060	2.95E-04	5.59E-08
**COG3256**	Nitric oxide reductase large subunit	Inorganic ion transport and metabolism	-3.775	4.241	3.05E-03	1.53E-05
**COG5314**	Conjugal transfer/entry exclusion protein	Mobilome: prophages, transposons	-3.698	7.229	6.07E-03	2.46E-04
**COG4292**	Low temperature requirement protein LtrA (function unknown)	Function unknown	-3.635	3.580	1.15E-03	3.74E-06
**COG4132**	ABC-type uncharacterized transport system, permease component	General function prediction only	-3.528	7.289	4.75E-03	2.48E-04
**COG4413**	Urea transporter	Amino acid transport and metabolism	-3.527	4.633	8.87E-03	3.16E-04
**COG4146**	Uncharacterized membrane permease YidK, sodium:solute symporter family	General function prediction only	-2.663	7.585	4.26E-04	1.62E-06
COG3542	Predicted sugar epimerase, cupin superfamily	General function prediction only	-2.640	4.961	8.87E-03	2.19E-04
COG2452	Predicted site-specific integrase-resolvase	Mobilome: prophages, transposons	-2.579	7.767	6.32E-03	1.97E-04
COG5520	O-Glycosyl hydrolase	Cell wall/membrane/envelope biogenesis	-2.201	9.260	6.32E-03	5.67E-04
COG4372	Uncharacterized conserved protein, contains DUF3084 domain	Function unknown	-1.888	10.155	1.24E-03	5.57E-05
COG0728	Peptidoglycan biosynthesis protein MviN/MurJ, putative lipid II flippase	Cell wall/membrane/envelope biogenesis	-1.621	9.419	8.71E-03	9.85E-04
COG1004	UDP-glucose 6-dehydrogenase	Cell wall/membrane/envelope biogenesis	-1.322	9.261	5.24E-03	6.10E-04
COG0627	S-formylglutathione hydrolase FrmB	Defense mechanisms	-1.241	8.546	3.17E-03	1.24E-04
COG0362	6-phosphogluconate dehydrogenase	Carbohydrate transport and metabolism	-1.118	9.309	5.19E-03	6.79E-04
COG0657	Acetyl esterase/lipase	Lipid transport and metabolism	-1.051	10.130	7.05E-03	6.64E-04
COG0110	Acetyltransferase (isoleucine patch superfamily)	General function prediction only	-0.885	9.518	6.32E-03	1.92E-04
COG0738	Fucose permease	Carbohydrate transport and metabolism	-0.780	10.289	6.64E-03	1.53E-04
COG1686	D-alanyl-D-alanine carboxypeptidase	Cell wall/membrane/envelope biogenesis	0.964	9.148	4.33E-03	7.24E-03
COG3887	c-di-AMP phosphodiesterase, consists of a GGDEF-like and DHH domains	Signal transduction mechanisms	1.201	7.785	8.87E-03	6.00E-03
COG3857	ATP-dependent helicase/DNAse subunit B	Replication, recombination and repair	1.239	8.504	4.71E-03	3.88E-03
COG0301	Adenylyl- and sulfurtransferase ThiI, participates in tRNA 4-thiouridine and thiamine biosynthesis	Coenzyme transport and metabolism	1.298	6.853	7.05E-03	5.84E-03
COG3290	Sensor histidine kinase regulating citrate/malate metabolism	Signal transduction mechanisms	1.377	9.331	1.60E-03	2.26E-03
COG4932	Uncharacterized surface anchored protein	Function unknown	1.412	11.054	8.60E-04	1.20E-03
COG0825	Acetyl-CoA carboxylase alpha subunit	Lipid transport and metabolism	1.423	6.612	3.75E-03	2.32E-03
COG1199	Rad3-related DNA helicase	Replication, recombination and repair	1.442	8.417	3.61E-03	6.21E-03
COG2357	ppGpp synthetase catalytic domain (RelA/SpoT-type nucleotidyltranferase)	Nucleotide transport and metabolism	1.444	6.705	3.05E-03	1.50E-03
COG4720	Uncharacterized membrane protein	Function unknown	1.509	6.663	1.12E-03	3.33E-04
COG1638	TRAP-type C4-dicarboxylate transport system, periplasmic component	Carbohydrate transport and metabolism	1.572	8.267	3.90E-04	7.41E-04
COG4109	Predicted transcriptional regulator containing CBS domains	Transcription	1.808	5.657	8.92E-04	3.78E-04
COG3688	Predicted RNA-binding protein containing a PIN domain	General function prediction only	1.824	6.121	6.09E-04	1.22E-04
COG1307	Fatty acid-binding protein DegV (function unknown)	Lipid transport and metabolism	1.856	7.624	9.16E-05	6.39E-05
COG3331	Penicillin-binding protein-related factor A, putative recombinase	General function prediction only	1.864	4.516	4.87E-04	1.18E-04
COG1001	Adenine deaminase	Nucleotide transport and metabolism	1.889	7.376	3.10E-03	3.36E-03
COG4753	Two-component response regulator, YesN/AraC family, consists of REC and AraC-type DNA-binding domains	Transcription	1.900	7.971	3.17E-03	4.88E-03
COG4709	Uncharacterized membrane protein	Function unknown	1.913	4.692	1.88E-03	9.03E-04
COG2179	Predicted phosphohydrolase YqeG, HAD superfamily	General function prediction only	1.935	4.166	6.40E-04	2.00E-04
COG1671	Uncharacterized conserved protein YaiI, UPF0178 family	Function unknown	1.975	5.369	4.75E-03	6.48E-03
COG1344	Flagellin and related hook-associated protein FlgL	Cell motility	2.007	8.124	3.95E-03	9.53E-03
COG0727	Fe-S-cluster containing protein	General function prediction only	2.009	5.909	6.19E-04	3.68E-04
COG1345	Flagellar capping protein FliD	Cell motility	2.022	7.356	3.75E-03	5.88E-03
COG4640	Uncharacterized membrane protein YvbJ	Function unknown	2.153	5.735	2.94E-03	2.87E-03
COG4717	Uncharacterized protein YhaN	Function unknown	2.199	5.526	1.97E-03	1.73E-03
COG3760	Uncharacterized protein	Function unknown	2.206	4.658	6.19E-04	5.54E-04
COG4862	Negative regulator of genetic competence, sporulation and motility	Transcription	2.301	4.990	3.05E-04	1.29E-04
COG2607	Predicted ATPase, AAA+ superfamily	General function prediction only	2.381	5.081	3.17E-03	4.87E-03
COG4728	Uncharacterized protein	Function unknown	2.486	3.669	4.87E-04	1.97E-04
**COG3108**	Uncharacterized conserved protein YcbK, DUF882 family	Function unknown	2.545	6.373	3.05E-04	6.34E-04
**COG1775**	Benzoyl-CoA reductase/2-hydroxyglutaryl-CoA dehydratase subunit, BcrC/BadD/HgdB	Secondary metabolites biosynthesis, transport and catabolism	2.592	6.286	4.54E-04	6.34E-04
**COG4509**	Uncharacterized protein	Function unknown	2.602	6.946	3.26E-05	1.31E-05
**COG1645**	Uncharacterized Zn-finger containing protein, UPF0148 family	General function prediction only	2.617	3.892	2.95E-04	1.62E-04
**COG4478**	Uncharacterized membrane protein	Function unknown	2.657	4.231	5.77E-05	1.53E-05
**COG1036**	Archaeal flavoprotein	Energy production and conversion	2.765	3.623	3.05E-04	2.45E-04
**COG4769**	Uncharacterized membrane protein	Function unknown	2.999	3.649	3.94E-05	2.44E-05
**COG4805**	Uncharacterized conserved protein, DUF885 family	Function unknown	3.142	5.282	2.84E-03	5.28E-03
**COG4223**	Uncharacterized conserved protein	Function unknown	3.541	4.149	1.46E-03	5.42E-03
**COG4939**	Major membrane immunogen, membrane-anchored lipoprotein	Function unknown	4.122	2.791	6.29E-06	5.08E-05

The abundance differences of 60 COGs that were statistically significant when comparing the formula-fed cohort with the breast-fed cohort are listed with COG category and an “example gene” description. Highlighted COGs represent top 10 most abundant COG in breast-fed (light gray) and formula-fed (dark gray) samples. LogFC values are relative to breast-fed abundance. Negative logFC indicates a genus was n fold lower in formula-fed compared with breast-fed; positive logFC indicates a genus was n fold higher in formula-fed compared with breast-fed.

**Figure 2:**
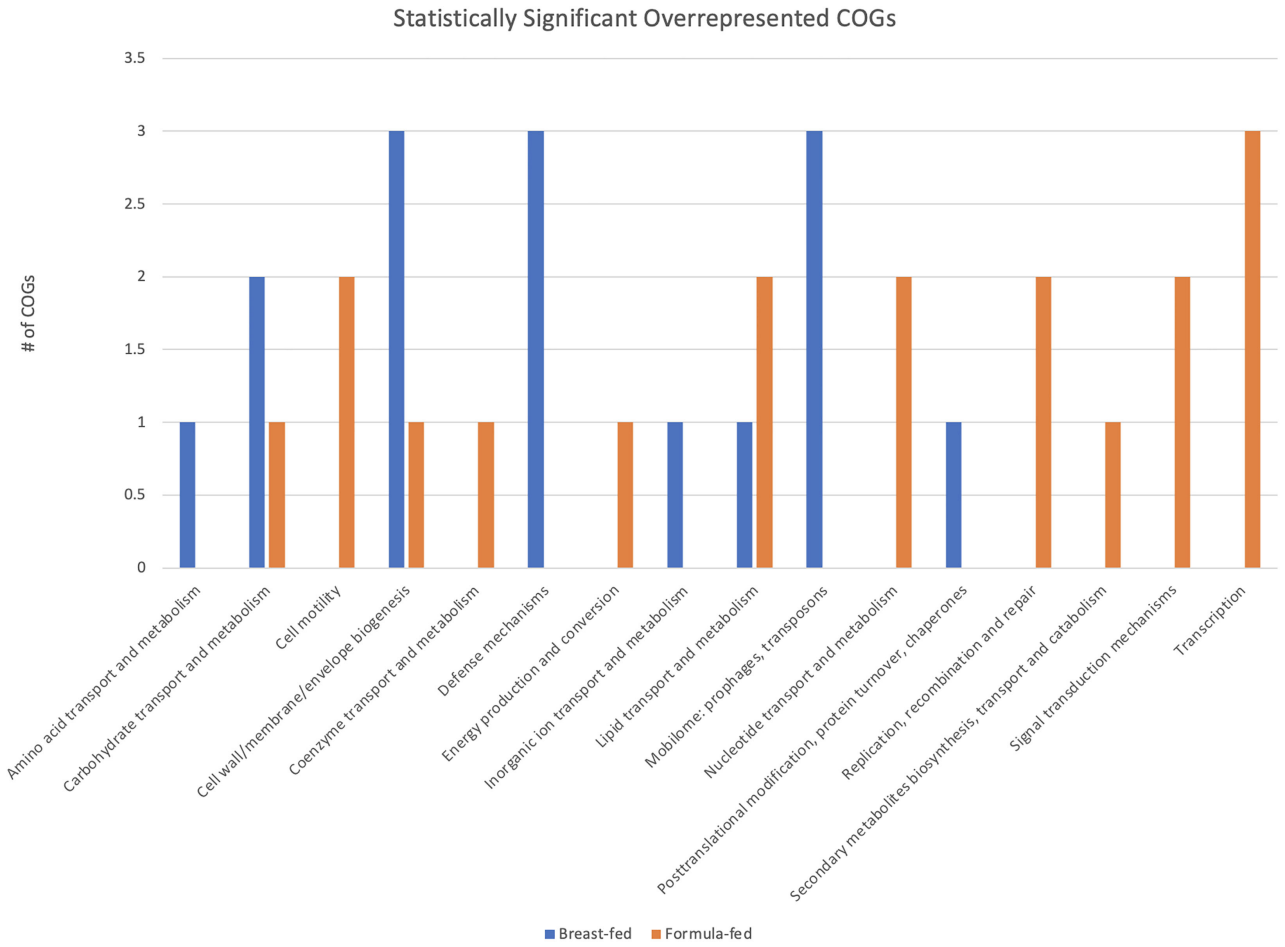
Clusters of Orthologous Genes (COG) Category Analysis. Statistically significant and overrepresented COGs are plotted by category and number from the total 60 COGs listed in **Table 2**.

A total of 21 COGs showed greater abundance in the BF group and 39 exhibited higher abundance in the FF group (**Figure 2**). Patterns of differences between the two cohorts for a variety of COGs were observed. Four COG categories were significantly overrepresented in BF samples: amino acid transport and metabolism, defense mechanisms, mobilome, and inorganic ion transport and metabolism. For FF samples, five categories were significantly overrepresented: cell motility; nucleotide transport and metabolism; replication, recombination, and repair; signal transduction mechanisms; and transcription.

**Figure 3:**
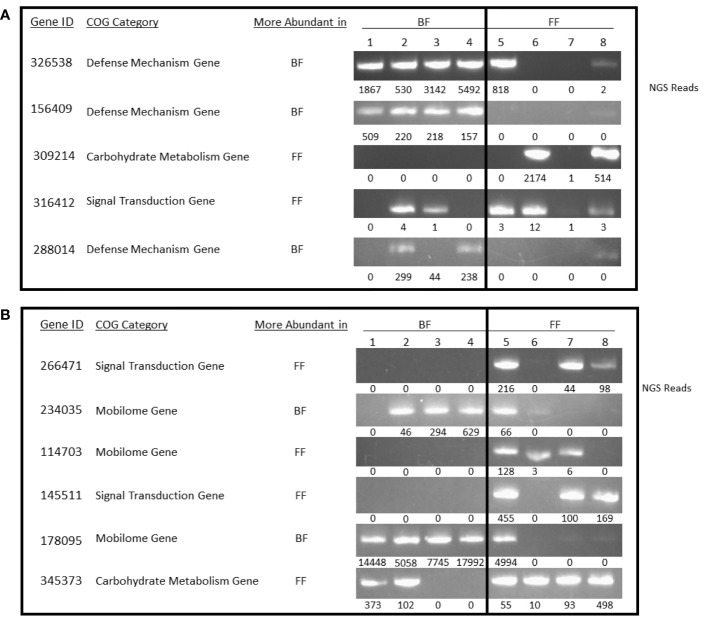
Gene Amplification. **(A, B)** Non-quantitative PCR was used to validate the results of the bioinformatic analysis for 11 genes. Four representative samples from each cohort of breast-fed and formula-fed infants were analyzed using the purified DNA extracts. Next generation sequencing reads are shown under each PCR panel, as well as corresponding cohort with a higher abundance of the Clusters of Orthologous Genes category. **(A)** 326538, toxin component of the Txe-Axe toxin-antitoxin module, Txe/YoeB family; 156409, alkyl hydroperoxide reductase subunit AhpF; 309214, glycogen synthase; 316412, c-di-AMP phosphodiesterase, consists of a GGDEF-like and DHH domains; 288014, S-formylglutathione hydrolase FrmB. **(B)** 266471, chemotaxis protein CheY-P-specific phosphatase CheC; 234035, conjugal transfer/entry exclusion protein; 114703, phage DNA packaging protein, Nu1 subunit of terminase; 145511, adenylate cyclase, class 3; 178095, protein involved in initiation of plasmid replication; 345373, sensor histidine kinase regulating citrate/malate metabolism.

The COGs that were overrepresented in each cohort were further analyzed using directed PCR to validate presence/absence of specific genes within each identified COG (**Figures 3A, B**). Specific primers were created to conduct gene/COG amplification to 11 additional genes within the COG categories that were suggested as having the most variance/difference between cohorts (defense mechanism, carbohydrate metabolism, signal transduction, and mobilome). Some genes were completely absent in the samples tested, as expected; others were amplified in both, though with incongruence between amplification and the NGS raw reads (**Figures 3A, B**). The PCR are end point and representative of differences between cohorts.

#### 3.3.1 Specific Genes

To evaluate and validate the shotgun metagenomics data, we conducted a polymerase PCR followed by gel electrophoresis on candidate markers identified. Four samples were selected from the BF cohort and four samples from the FF based on sample availability for validations. The number of NGS reads supporting the abundance for a given gene and sample are displayed under the gel band. **Figure 3A** focuses on five genes identified in the shotgun metagenomics analysis that are related to defense mechanisms, carbohydrate metabolism, or signal transduction pathways. As demonstrated in the gel pictures, the predicted abundances from the NGS data correlated with the NGS read counts. Gene ID 156409 was the only gene in which there was a perfect absence (FF) and presence (BF) between cohorts. Consistent with other metagenomic publications (Baumann-Dudenhoeffer et al., 2018) it is not uncommon to detect variability within a cohort, even when statistically significant differences are noted at the population level (BF vs FF).

**Figure 3B** focuses on six genes identified in the shotgun metagenomics analysis that are related to signal transduction pathways, mobilome, and carbohydrate metabolism. Like the genes analyzed in **Figure 3A**, the predicted abundances from the NGS data correlated with the NGS read counts. **Supplemental Table 2** contains all the analyzed subjects’ shotgun metagenomics NGS read count data.

## 4 Discussion

The metagenomic analysis presented here demonstrates a robust and useful tool for analyzing fecal microbiomes early in life. In the present study, a larger cohort expands the sensitivity of the analysis from prior work (Di Guglielmo et al., 2019) and allows a more in-depth analysis of COG and gene differences in the gut microbiome between the two cohorts, FF and BF, of young infants. Understanding whether these differences are biologically significant, and whether they are permanent through childhood, remain goals of both this study and a future longitudinal study. The Bray-Curtis PCoA clustering implies an abundance of species separation between cohorts, further reinforcing that infant feeding even at early ages influences the gut microbiota contrast and congruence.

### 4.1 Metataxonomic Trends and Differences

The diversity and differentiation between cohorts is represented, both summated, and by individual subject, in **Supplemental Figure 1**. A wider distribution of diversity is seen in the BF cohort; however, the index distribution values are lower compared with the FF cohort. While some subjects stand out as unique, the overall trend represents diversity differentiation, with the FF cohort having a greater diversity (overall Shannon diversity index approaching statistical significance). Specifically, one subject in the FF cohort had a very high abundance of *Bacteroides*, which was almost uniformly seen in the BF cohort with increased abundance, likely skewing the Shannon diversity index. A greater diversity in FF infants has been associated with poorer health outcomes and dysbiosis and is contrasted by a lower diversity in BF infants (Schwartz et al., 2012; Savage et al., 2018; Davis et al., 2020).

Thirty-nine genera are differentially represented between cohorts, with a similar pattern to our prior work (Di Guglielmo et al., 2019). Of note, between our prior reported cohorts and our expanded cohort, the genera with statistical significance between cohorts (**Figure 1A**, **Supplemental Table 4**) are consistent: *Parabacteroides*, *Haemophilus*, *Clostridioides*, and *Staphylococcus*. In the expanded cohort, *Bifidobacterium*, *Enterococcus*, and *Lachnoclostridium* are also statistically significantly different between cohorts. Breast-fed infants have greater relative abundance of *Bifidobacterium*, a gram-positive bacterium of phylum *Actinobacteria*, used in many probiotics. As expected, the *Bifidobacterium* genus is highly abundant in the BF cohort consistent with prior reports (Henrick et al., 2018; Karav et al., 2018; Henrick et al., 2019). *Bifidobacterium* is strongly associated with breast milk feeding and is therefore expected to be found in the infant gut. *Lachnoclostridium*, a microbe in the phylum *Firmicutes*, but of unknown pathogenic potential, is more abundant in the FF cohort. The greater abundance in the FF cohort of *Enterococcus* may also reflect a more pathogenic-potential microbial shift in the gut of these infants. The trend bears further investigation in terms of both gene abundance and metabolic output to determine clinical and biological significance for these infants (Fukuda et al., 2012; den Besten et al., 2013). Caesarean section delivery was more frequent in the FF infants; however, the mode of delivery influences the infant microbiota/microbiome (Rutayisire et al., 2016; Korpela, 2021). In the cohort genera metataxonomic analysis (**Figure 1A**), data show that *Bifidobacterium* is comparable between cohorts, while *Klebsiella* and *Bacteroides* are not. *Escherichia* was similar between cohorts, while *Veillonella* was not. While differences between delivery modes have been studied, the results in this cohort are not completely aligned with prior studies. To ascertain whether the influence on taxonomy alone of delivery is enough to cause cohort differences, the more in-depth analysis available with whole-genome metagenomics offers an advantage.

### 4.2 Metagenomic Observations and Implications

Sixty COGs were significantly more abundant in either BF or FF infants (**Table 2**). Examining those COGs that are significantly overrepresented in either FF or BF cohorts (**Figure 2**) reveals some notable patterns. There are COGs with specific functions that are absent or present in each of the cohorts. Should these COGs and the protein domains they represent result in the loss or gain of protein function, the abundance differences may influence metabolically active, beneficial, or pathologic proteins within the gut. It is not yet clear if these changes are permanent or whether they contribute to intra- or extra-intestinal disorders in infancy and childhood.

Using the difference in abundance of these 60 COGs as informed by the heatmap (**Supplemental Figure 3**), we focused on four COGs: carbohydrate transport and metabolism; defense mechanisms; mobilome—prophages, transposons; and signal transduction mechanisms (**Figure 2**). In our prior work, we observed some genes that were present only, or predominantly, in one cohort vs. the other, namely CRISPR-Cas9 and carboxypeptidase (Di Guglielmo et al., 2019).

For the current study, specific genes from each COG demonstrate similar patterns as the NGS read counts and shows alignment with abundance data (**Figure 3**). Five out of five genes more abundant in the BF samples analyzed are entirely or almost entirely absent from the FF samples. Four genes (309214, 266471, 114703, 145511) that are more abundant in FF infants are completely absent in the BF samples analyzed; two genes (316412, 345373) that are more abundant in FF infants are mostly absent from the BF samples with two BF samples detected and two BF samples not detected (**Figure 3**; **Supplemental Table 2** contains the supporting NGS read count data). Of the genes completely absent in our samples from either cohort, the pattern indicates that defense mechanism genes are absent (two of three) in FF samples while a carbohydrate metabolism gene, two signal transduction genes, and a mobilome gene are absent in some BF samples. For the defense mechanism genes, this indicates that FF infant microbiota may lack the ability to thwart DNA level changes that could confer greater susceptibility to mutative changes. The implication is the potential in FF infants for the introduction of pathologic or inflammatory proteins affecting gut immune stability and overall homeostasis (Fallani et al., 2010; Guaraldi and Salvatori, 2012; Schwartz et al., 2012; Turroni et al., 2020; Sarkar et al., 2021). For the BF infant microbiota, some of the genes in these categories may be associated with reduced propensity for metabolic shifts that confer dysbiosis or altered signal transduction. Regarding mobilome genes, a loss or gain of horizontal gene transfer through mobile genetic elements (Jørgensen et al., 2015; Mancino et al., 2019; Carr et al., 2021), related to selection pressure-driven changes, may facilitate or impede antibiotic resistance in the differently fed cohorts.

Based on the function of these genes, and what they contribute to in terms of either signaling, carbohydrate metabolism, or health of the gut bacteria themselves, we may be able to examine impact on metabolic (obesity), immunologic (antibiotic-resistance), and other long-term health issues. Does a more dysbiotic young infant gut lead to cellular stress or inflammation that interferes with gut signaling? This is a hypothesis that warrants further study. The gut may normalize over time based on later infancy and toddlerhood diets such that there is no permanent impact. Conversely, these microbial influences and changes may be more permanent (Turroni et al., 2020; Sarkar et al., 2021) because of the overabundance or underabundance of certain key organisms, the genes they express, and the proteins those genes encode. Ongoing work in our laboratory aims to demonstrate short-chain fatty acid differences that exist between cohorts as well as longitudinal taxonomic and genera/COG/gene abundance differences (Fukuda et al., 2012; den Besten et al., 2013).

### 4.3 Limitations

There are both strengths and limitations to the present study. Shotgun metagenomics enables a non-biased approach that can yield gene level data compared with 16S methods. This type of method requires extensive bioinformatic pipelines (Di Guglielmo et al., 2019) and generates data that enables gene level analysis that can be utilized for functional interpretation, which is not possible with the traditional 16S approach. The present study has a larger sample size compared with our previous report. Limitations include that our FF cohort is less than half the sample size as our BF cohort, and thus outliers with different taxonomy or metagenomes/COG could affect both statistical significance and definitiveness of conclusions. The CRISPR-Cas9 gene from our prior work was detected in samples in this study but the abundance difference was not statistically significant, reflecting the smaller FF cohort size. Of note, this gene is known to be variable in different taxonomies (Crawley et al., 2018). Yet some differences are striking enough to be worth reporting and pursuing further. We cannot control for the maternal or environmental microbiome and their impact/influence on even very young infants, as we see no clear pattern in presence/absence of genera of individual subject’s sample regardless of sex and age at sample collection or delivery method. We acknowledge that delivery method was statistically different between cohorts (**Table 1**), which may present a confounding variable. In prior studies, with small cohorts, Caesarean section delivery was either an exclusion criterion with no data was presented to make a comparison (Lee et al., 2015) or not mentioned at all (Schwartz et al., 2012). We note that in our study, within a cohort, there was variability in the relative abundances of genera between samples from babies born *via* vaginal delivery and between samples from babies born *via* caesarean section delivery. Nonetheless, the data do show that there are notable summative differences in the cohorts. In a potential future study with larger sample size, a covariate regression analysis could be completed to ascertain the role of delivery; however, in our study our sample size is not large enough to make definitive statements about the impact of delivery. Our group has banked additional samples from infants not enrolled in this study who had the same dichotomous feeding method but did not meet all inclusion criteria. Using the analysis pipeline, and looking for similar patterns of abundance, diversity, variance, and COG overrepresentation, we may be able to elucidate patterns for each cohort beyond the strict criteria used for this study. Longitudinal microbiome data from additional collected samples on these patients will allow us to answer questions about whether these early changes and differences are temporary or permanent. Finally, metabolic data on these samples, currently under analysis, will demonstrate, potentially, unique characteristics by subject and cohort.

The present study demonstrates key differences between BF and FF cohorts in both taxonomic signature of the gut microbiota and metagenome. Several defense mechanism genes are virtually absent in the FF cohort, as confirmed by PCR validation. Gut bacteria in these infants, if more susceptible to phage, virus, and other transformative changes due to defense mechanism gene absence, could lead to pathogenicity and/or specific dysbiotic characteristics such as antibiotic resistance. With these changes can come inflammation and other cellular stressors that alter the ability of both microbiota, and host, to maintain homeostasis. Regardless of whether the gut microbiome signature normalizes later in life, the impact early on of gene abundance (or absence) as a proxy for function cannot be ignored. This research may lead to solutions for restoring gut health in FF infants, or those exposed to antibiotics at an early age, with the intent to boost the abundance of bacteria (Underwood et al., 2013; Karav et al., 2018; Henrick et al., 2019) that return specific proteins and their function to the gut.

## Data Availability Statement

The datasets presented in this study can be found in online repositories. The names of the repository/repositories and accession number(s) can be found below: https://www.ncbi.nlm.nih.gov/bioproject/?term=PRJNA542703 and https://www.ncbi.nlm.nih.gov/bioproject/?term=PRJNA789149.

## Ethics Statement

The studies involving human participants were reviewed and approved by Nemours Institutional Review Board, Nemours Children’s Health, Wilmington, Delaware. Written informed consent to participate in this study was provided by the participants’ legal guardian/next of kin.

## Author Contributions

MD, KF, and EC contributed substantially to the conception or design of the work and the acquisition, analysis, or interpretation of data for the work. MD, KF, AR, and EC drafted the work and revised it critically for important intellectual content. MD, KF, AR, and EC provided approval for publication of the content and agree to be accountable for all aspects of the work in ensuring that questions related to the accuracy or integrity of any part of the work are appropriately investigated and resolved.

## Funding

Research Excellence program grant from the National Institute of General Medical Sciences of the National Institutes of Health under grant number P30 GM114736 (PI: Shaffer) and by an Institutional Development Award (IDeA) from the National Institute of General Medical Sciences of the National Institutes of Health under grant number U54 GM104941 (PI: Binder-Macleod).

## Conflict of Interest

The authors declare that the research was conducted in the absence of any commercial or financial relationships that could be construed as a potential conflict of interest.

## Publisher’s Note

All claims expressed in this article are solely those of the authors and do not necessarily represent those of their affiliated organizations, or those of the publisher, the editors and the reviewers. Any product that may be evaluated in this article, or claim that may be made by its manufacturer, is not guaranteed or endorsed by the publisher.

## Supplementary Material

Supplementary Figure 3 Heat map of differentially represented COGs.

## Abbreviations

COG, Clusters of Orthologous Genes; BF, breast-fed; FF, formula-fed; NGS, next generation sequencing.
