# Supplementary figures and images for: Impact of Early Feeding: Metagenomics Analysis of the Infant Gut Microbiome
Source: Front Cell Infect Microbiol. 2022 Mar 4;12:816601. doi: 10.3389/fcimb.2022.816601 (PMC8931315; doi:10.3389/fcimb.2022.816601)

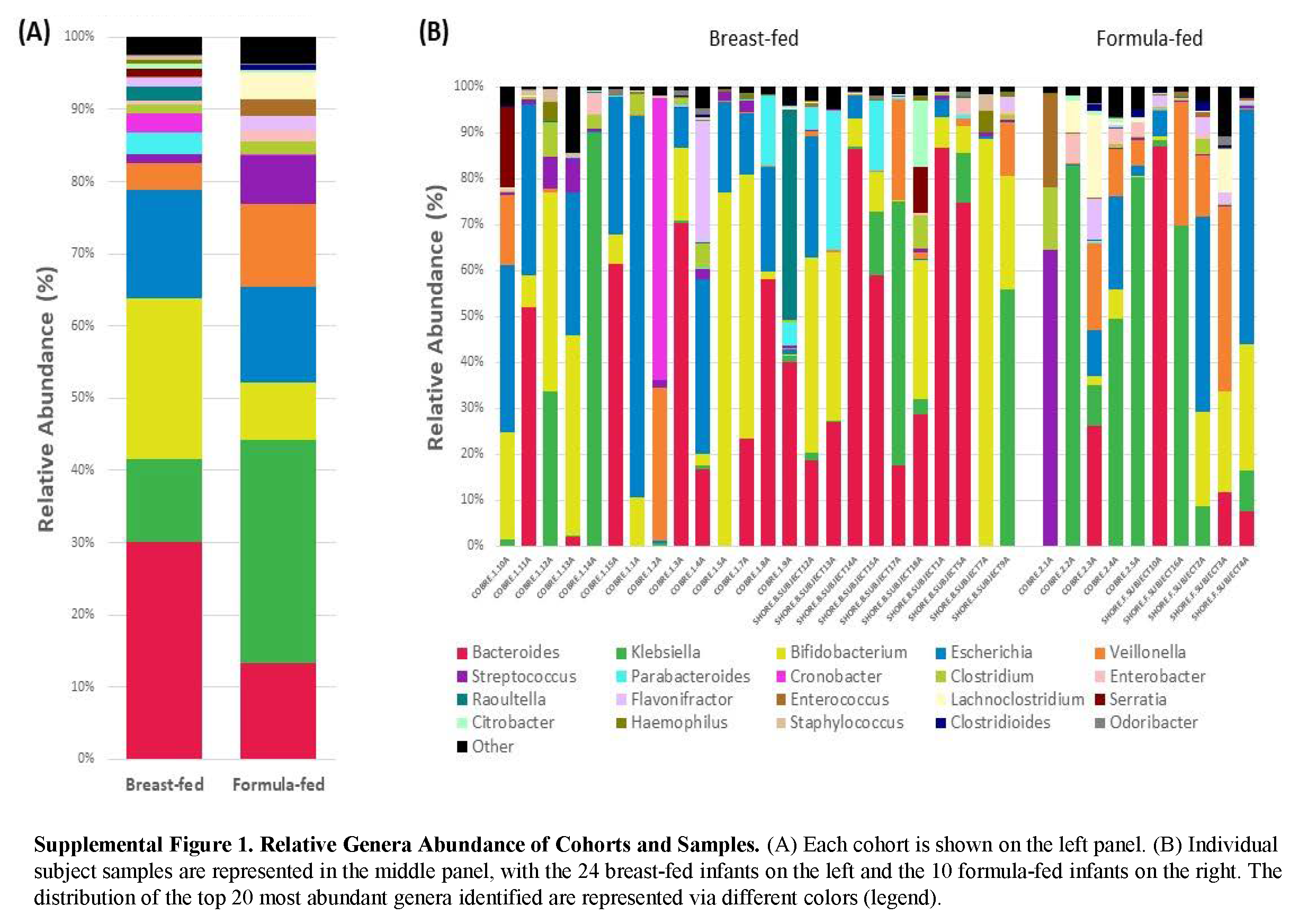

Supplement: Supplementary file 1 [file Image_1.tif]

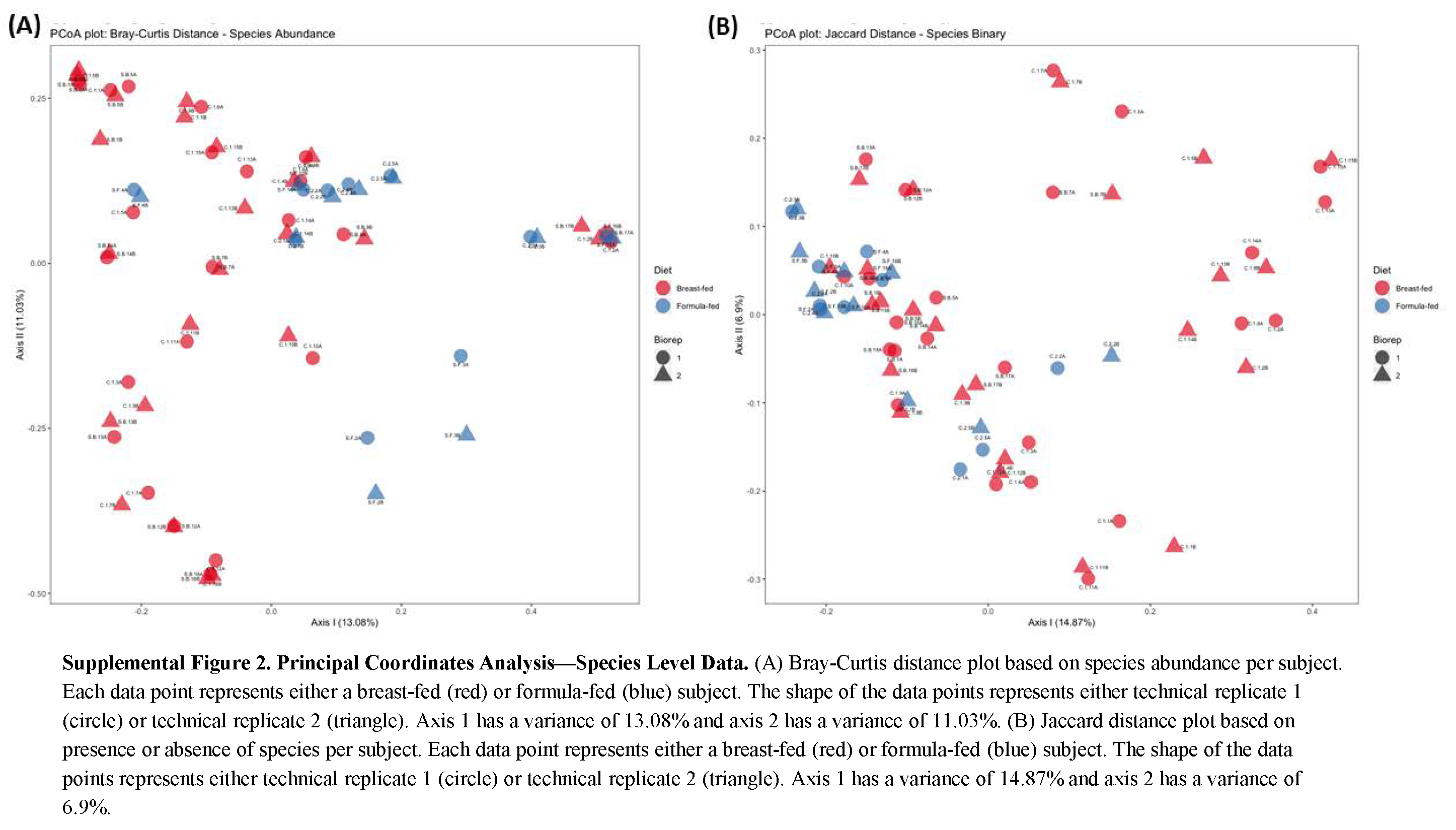

Supplement: Supplementary file 2 [file Image_2.tif]

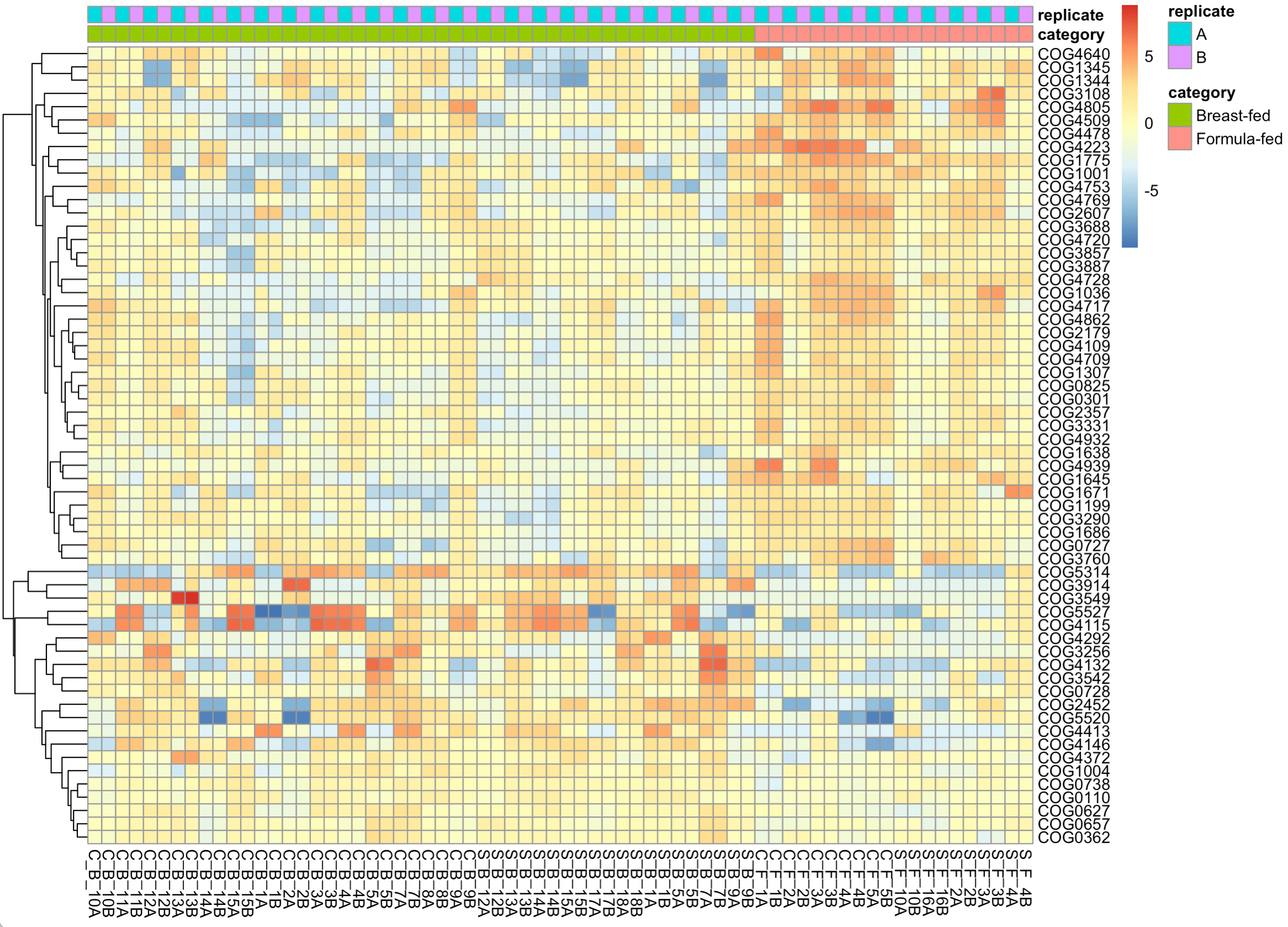

Supplement: Supplementary Figure 3 — Heat map of differentially represented COGs. [file Image_3.tiff]
